# Electronic Cigarette Exposure Induces Adverse Cellular Alterations in Skeletal Muscle in Male Mice Subjected to a High-Fat Diet

**DOI:** 10.3390/ijms262311491

**Published:** 2025-11-27

**Authors:** Juan Carlos Rivera, Jorge Espinoza-Derout, Kamrul Hasan, Candice J. Lao, Julian Wilson, Yin Tintut, Xuesi M. Shao, Maria C. Jordan, Kenneth P. Roos, Yanjun Liu, Amiya P. Sinha-Hikim, Vishwajeet Puri, Theodore C. Friedman

**Affiliations:** 1 Division of Endocrinology, Metabolism and Molecular Medicine, Department of Internal Medicine, Charles R. Drew University of Medicine and Science, 1731 E. 120th Street, Los Angeles, CA 90059, USA; jorgeespinozaderout@cdrewu.edu (J.E.-D.); mshao@g.ucla.edu (X.M.S.);; 2 Department of Medicine, David Geffen School of Medicine, University of California, Los Angeles, CA 90095, USA; 3 Department of Physiology, David Geffen School of Medicine, University of California, Los Angeles, CA 90095, USA; 4 Department of Biomedical Sciences and Diabetes Institute, Ohio University Heritage College of Osteopathic Medicine, Ohio University, Athens, OH 45701, USA; puri@ohio.edu

**Keywords:** electronic cigarette, nicotine, skeletal muscle, high-fat diet, obesity

## Abstract

Electronic cigarettes (E-Cig) are a new way of delivering nicotine, gaining popularity among adolescents and young adults, who often do not realize their harmful effects. Although the adverse effects of E-Cigs on the liver and heart have been demonstrated, their effects on the skeletal muscle have not been well studied. In this study, we evaluated the skeletal muscle effects of E-Cig aerosol, delivered in a manner similar to human vaping, in a mouse model of obesity induced by a high-fat diet (HFD). C57BL/6 mice, fed either a normal chow diet (NCD) or HFD, were exposed to either saline aerosol control or aerosol generated from Blu PLUS^TM^ containing 0% or 2.4% nicotine for 12 weeks. Mice fed an NCD were included to distinguish whether E-Cig effects on the skeletal muscle required the presence of obesity induced by an HFD. The soleus muscle, an oxidative muscle rich in mitochondria, was assessed by Western blotting, electron microscopy, and biochemical assays. An NCD group was included to assess the baseline effects of HFD-induced obesity, on the skeletal muscle. The skeletal muscle from HFD-fed mice exposed to E-Cig 2.4% had reduced levels of phospho-AMPK compared with saline and E-Cig 0% groups, while E-Cigs had no effect on NCD-fed mice. Levels of phospho-adipose triglyceride lipase were also reduced in both E-Cig 2.4% and 0% compared with the saline group. These metabolic protein impairments were accompanied by increased levels of oxidative stress and phospho-p38 MAPK. Deregulation of the autophagy markers, microtubule-associated protein 1A/1B-light chain 3 (LC3-I; inactive form) and LC3-II (active form), was also observed, evidenced by decreased levels of LC3-II, ratio LC3-II/LC3-I, and increased levels of p62. Transmission electron microscopy analysis showed that E-Cig 2.4% induced damage to mitochondrial structure compared with the saline or E-Cig 0% groups. These findings suggest that E-Cig exposure on HFD impairs the skeletal muscle, adding to the growing list of affected organs for ongoing regulatory efforts concerning nicotine-containing substances.

## 1. Introduction

Cigarette smoking continues to be one of the principal causes of morbidity and mortality in the United States (USA) [1]. Electronic cigarettes (E-Cig) are a new way of delivering nicotine. E-Cigs have gained popularity quickly since their introduction due to attractive devices and flavors [2]. In the USA, the principal tobacco product consumed by adolescents and young adults is E-Cig, and these consumers often do not realize the harmful health effects of E-Cig [3,4]. It is alarming that 5.9% of middle and high school adolescents reported using an E-Cig in the last 30 days [4]. In fact, E-Cig consumption in young adults (18–24 years) increased from 2.7% in 2017 to 10.3% in 2023 and in adults (25–44 years) from 1.5% in 2017 to 6.1% in 2023 [5]. These trends pose a national health problem, affecting a wider age range of E-Cig users, from those aged 18 to 44. In addition, nicotine, one of the principal components of the E-Cig, is a highly addictive substance [6]. In animal models fed a high-fat diet (HFD), E-Cig has been shown to produce detrimental consequences on metabolic functions, including hepatic steatosis [7] and cardiac dysfunction [8], highlighting the widespread detrimental molecular consequences of E-Cig use.

Obesity, which increases susceptibility to chronic diseases, is a second risk factor affecting 22% of adolescents and the young adult population [9,10]. Interestingly, E-Cig use is increasingly more common in overweight boys [11,12] and girls, who are trying to lose weight [13], possibly due to the perception that E-Cig helps with weight loss [14]. As obesity further reduces physical activity [15] due to discomfort and fatigue [16], adolescents who use E-Cig are less engaged in moderate-to-vigorous physical activities compared with never-users [17], further affecting their quality of life [18].

The skeletal muscle is essential for maintaining a healthy quality of life, enabling physical activity and reducing the risk of metabolic disease and chronic diseases [19]. To maintain skeletal muscle health, cellular and molecular mechanisms such as autophagy, oxidative stress, and mitochondria need to work in concert [19,20]. Skeletal muscle dysfunction has debilitating consequences, such as reduced muscle mass and strength, which are associated with fragility and chronic diseases [20,21]. Since the principal consumers of E-Cig are adolescents and young adults with ongoing skeletal muscle maturity, its atrophy would pose long-term adverse health consequences.

In humans, in addition to causing pulmonary diseases, cigarette smoking adversely affects skeletal muscle atrophy, including quicker muscle fatigue, reduction in exercise performance, muscle atrophy, decreased ATP production, and regenerative capacities, as well as an increase in fat infiltration, oxidative stress, and phosphorylated p38 mitogen-activated protein kinase (p-p38 MAPK) [22]. Preliminary data from the European Respiratory Society recently reported that human E-Cig users had reduced exercise capacities compared to non-smokers and similar to that of conventional cigarette users [23]. In animal models, chronic E-Cig exposure (4 months) reduced treadmill performance and ex vivo strength [24], and short-term E-Cig exposure (14 days) reduced grip strength and endurance performance (swimming) [25]. These reports outline the negative consequences of E-Cig use on the skeletal muscle; however, the molecular pathways of these effects are still unknown. We have previously demonstrated that in HFD-fed mice, intraperitoneal (ip) injections of nicotine (two-hit model by using two risk factors, nicotine and HFD) cause gastrocnemius myofiber disorganization, intramyocellular lipid deposition, intramyofibrillar mitochondrial damage with increased oxidative stress, and a reduction in AMP-activated protein kinase (AMPK) phosphorylation [26]. In the present study, using a mouse model of HFD-induced obesity, we tested the hypothesis that nicotine delivered via E-Cig caused skeletal muscle abnormalities and elucidated its cellular and molecular mechanisms. Control groups, normal chow diet (NCD) ± E-Cig, were also included to assess whether E-Cig effects were only in the presence of HFD-induced obesity. These findings provide significant insights for public health as metabolic disease is a growing epidemic in Western countries.

## 2. Results

### 2.1. Effects of E-Cig Exposure on AMPK and ATGL Phosphorylation

**Figure 1:**
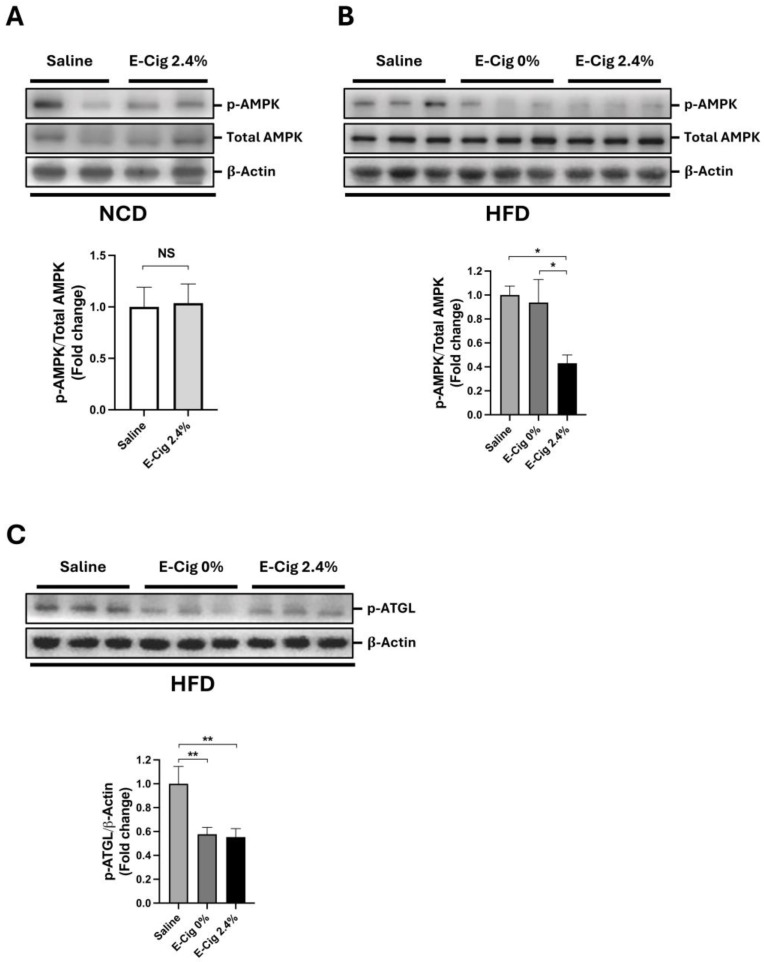
E-Cig exposure decreases AMPK and ATGL activation only in HFD-fed mice. (**A**) Representative Western blot images and quantitation for phosphorylated AMPK (p-AMPK) in skeletal muscle of mice fed NCD exposed to saline (*n* = 4) and E-Cig 2.4% (*n* = 4). (**B**) Mice were fed HFD and exposed to saline (*n* = 5), E-Cig 0% (*n* = 9), or E-Cig 2.4% (*n* = 10). Total AMPK levels were used as a loading control and normalization. (**C**) Representative Western blot images and quantitation for phosphorylated ATGL (p-ATGL). β-Actin levels are shown as a loading control. The protein levels were normalized to β-Actin. Molecular weight markers are depicted in KDa. The graphs are expressed as the mean ± S.E.M. (fold-change relative to the control). NS = not significant, * *p* < 0.05, ** *p* < 0.01.

AMPK is a key regulator of cellular metabolism in the skeletal muscle, and its phosphorylation (p-AMPK) state is crucial to adapt the energy requirements for physical activity [27]. First, we assessed the levels of p-AMPK in NCD-fed mice exposed to saline aerosol (control group) or E-Cig 2.4% (E-Cig with 2.4% nicotine). Western blot analysis showed that the p-AMPK levels were not significantly different (*p* = 0.09) between the control and E-Cig-exposed groups (Figure 1A). In HFD-fed mice, the p-AMPK levels were significantly lower in the skeletal muscle of mice exposed to E-Cig 2.4% nicotine compared with those exposed to E-Cig 0% nicotine (*p* = 0.04) or saline groups (*p* = 0.04) (Figure 1B).

We also measured skeletal muscle triglyceride levels in the groups fed NCD and HFD. Triglycerides levels were increased in all HFD groups compared with the saline plus NCD group (vs. saline + HFD *p* = 0.02, vs. E-Cig 0% + HFD *p* = 0.02, and E-Cig 2.4% + HFD *p* = 0.002) (Supplementary Figure S2). A significant increase in triglyceride levels was also detected in the E-Cig 2.4% plus HFD group compared with the E-Cig 2.4% plus NCD group (*p* = 0.02) (Supplementary Figure S2). Since p-AMPK and triglyceride levels were not significantly different in the NCD ± E-Cig groups, we continued our analysis only with the HFD groups.

Adipose triglyceride lipase (ATGL) is a downstream target of p-AMPK [28], which is phosphorylated at serine^406^ (p-ATGL). ATGL is the principal lipolysis enzyme that breaks down triglycerides in the skeletal muscle [29] and is required for lipolysis [29]. The results showed that serine^406^ phosphorylation of ATGL was lower in groups exposed to E-Cig (0% and 2.4%) compared with the saline group (*p* = 0.007 and *p* = 0.006, respectively) (Figure 1C). The results suggest that when combined with an HFD, E-Cig exposure reduced the activation of the skeletal muscle.

### 2.2. Effects of E-Cig Exposure on Oxidative Stress Markers

**Figure 2:**
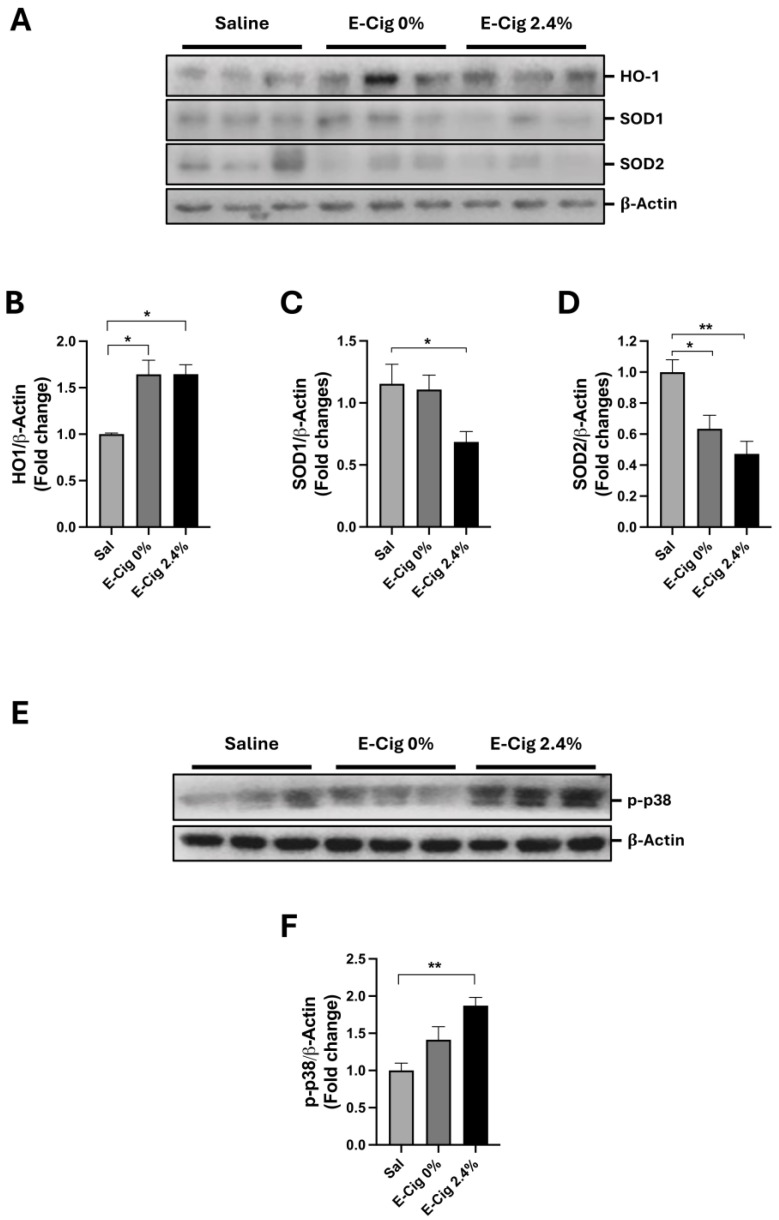
E-Cig exposure triggers oxidative stress and stress pathway response in the skeletal muscle. Representative Western blot images for HO-1, SOD1, and SOD2 in (**A**) skeletal muscle of mice fed HFD and exposed to saline (*n* = 4–5), E-Cig 0% (*n* = 9), or E-Cig 2.4% (*n* = 10). β-Actin is used as a loading control. Molecular weight markers are depicted in KDa. (**B**–**D**) Quantitation of protein levels for HO-1, SOD1, and SOD2 normalized to β-Actin. Representative Western blot images for (**E**) phospho-p38 MAPK (p-p38). β-Actin levels are shown as a loading control. Molecular weight markers are depicted in KDa. (**F**) Quantification of the protein levels for p-p38 were normalized to β-Actin and expressed as the mean ± S.E.M. (fold-change relative to the control). * *p* < 0.05, ** *p* < 0.01.

Heme oxygenase-1 (HO-1) is activated in response to oxidative stress [30], and superoxide dismutase 1 (SOD1) and superoxide dismutase 2 (SOD2) are two antioxidant enzymes that are reduced in high oxidative stress conditions [31]. We and others have previously shown that nicotine injections (ip) and HFD in mice increased oxidative stress in the skeletal muscle [26]. Thus, we assessed the muscle protein levels of HO-1, SOD1, and SOD2 in response to E-Cig exposure. The results showed that HO-1 levels were higher in the E-Cig groups compared with the saline group (*p* = 0.02 and *p* = 0.02, respectively) (Figure 2A,B). In contrast, SOD1 and SOD2 levels were significantly reduced in E-Cig 2.4% (*p* = 0.05 and *p* = 0.002, respectively) (Figure 2A,C,D). E-Cig 0% also reduced SOD2 levels compared with the saline group (*p* = 0.01) (Figure 2A,D). These results suggest that E-Cig exposure increases oxidative stress in the skeletal muscle.

### 2.3. Effects of E-Cig on Cellular Stress Pathway

In the skeletal muscle, oxidative stress triggers stress pathways, such as the activation of p38 MAPK (p-p38) by phosphorylation [32]. Thus, we assessed the phosphorylation of p38 MAPK in response to E-Cig exposure. The results showed that the E-Cig 2.4% group had significantly higher levels of p-p38 compared with the saline group (*p* = 0.006) (Figure 2E,F). This result suggests that only E-Cig 2.4% triggers a stress signal in skeletal muscle cells.

### 2.4. Effects of E-Cig Exposure on Autophagic Proteins LC3B

**Figure 3:**
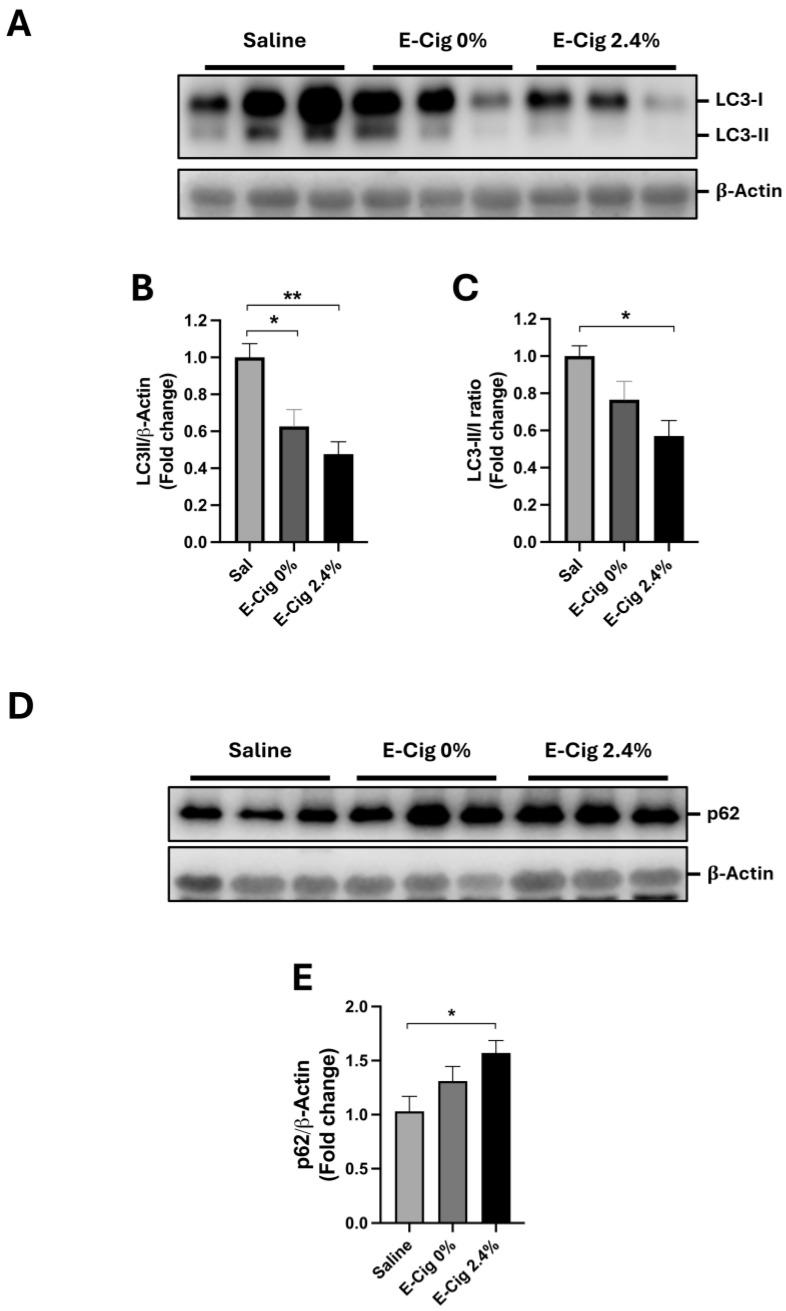
E-Cig exposure reduces LC3-II and LC3-II/LC3-I ratio in the skeletal muscle. Representative Western blot images for LC3B in (**A**). (**B**,**C**) LC3-II/LC3-I ratio and LC3-II protein levels were quantified. (**D**) Representative Western blot images for p62. β-Actin levels are shown as a loading control. Molecular weight markers are depicted in KDa. (**E**) Quantification of the protein levels of p62 normalized to β-Actin. The graphs are normalized to β-Actin and expressed as the mean ± S.E.M. (fold-change relative to the control). * *p* < 0.05, ** *p* < 0.01.

Autophagy maintains skeletal muscle cell homeostasis by removing unwanted organelles and proteins [33]. Alterations in the skeletal muscle, such as inactivation of AMPK, are associated with the deregulation of autophagy, increased oxidative stress [19,33], and activation of p38 MAPK [33]. Thus, we measured the protein levels of the key marker of autophagy, LC3B (LC3-I and LC3-II), in response to E-Cig exposure. LC3B is found in two forms, the inactive form (LC3-I) and the active form (LC3-II) [33]. As shown in Figure 3A,B, the levels of LC3-II (active form), but not LC3-I (inactive form), were significantly reduced in both E-Cig groups (0 and 2.4% nicotine) compared with the saline group (*p* = 0.02 and *p* = 0.001, respectively). The LC3-II/LC3-I ratio, the indicator of autophagic flux (degradation rate through the autophagy), was lower only in the E-Cig 2.4% group compared with the saline group (*p* = 0.02) (Figure 3C).

We also determined the protein levels of the main protein cargo of the autophagy, p62 [33], by Western blotting. The results showed that the p62 levels were significantly higher in the E-Cig 2.4% compared with the saline group (Figure 3D,E). Altogether, these results suggest that E-Cig 2.4% reduces the autophagy function in the skeletal muscle.

### 2.5. Effects of E-Cig on Mitochondrial Morphology (TEM)

**Figure 4:**
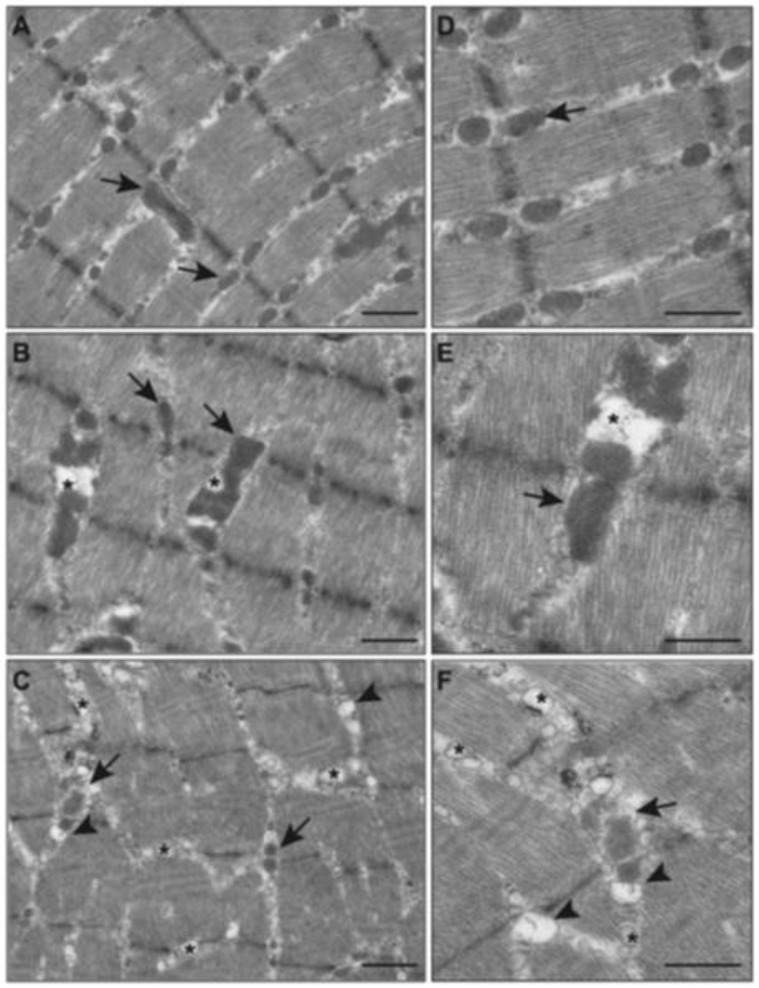
E-Cig exposure induces mitochondrial skeletal muscle damage. Representative transmission electron microscopic images shown in lower magnification ((**A**–**C**), scale bar = 800 nm) and in higher magnification ((**D**–**F**), scale bar = 600 nm) in the skeletal muscles of HFD-fed mice exposed to saline (**A**,**D**), E-Cig 0% (**B**,**E**), or E-Cig 2.4% (**C**,**F**). Arrows indicate the intermyofibrillar mitochondria, arrowheads indicate broken cristae in mitochondria, and the asterisks indicate IMCL accumulation.

We previously demonstrated that nicotine plus HFD altered mitochondrial morphology in the skeletal muscle of mice [26,34]. Mitochondrial damage accompanied following the changes in the reduced activation of AMPK and ATGL, increased oxidative stress and p-p38, and the deregulation of autophagy. Thus, we analyzed the ultrastructure of mitochondria by electron microscopy in mice exposed to E-Cig. In the control group (saline), mitochondria showed a normal structure with a well-defined sarcomeric and myofibrillar pattern as well as a normal intramyofibrillar (IMF) pattern (arrow, Figure 4A,D). In the group exposed to E-Cig 0%, the cellular architecture was similar to that of the saline group except for a few intramyocellular lipid (IMCL) deposits (asterisk, Figure 4B,E). In the group exposed to E-Cig 2.4%, there were relatively smaller, vacuolated mitochondria, cristolysis, and extensive IMCL accumulation (asterisk and arrowhead, Figure 4C,F). These results suggest that E-Cig 2.4% increases mitochondrial damage and lipid deposition in the skeletal muscle.

## 3. Discussion

This present study integrated signaling, redox, degradative, and ultrastructural endpoints to assess the effects of E-Cig in a two-hit model (HFD + nicotine) on skeletal muscle health. Although the prior study [26] using ip injections of nicotine ± HFD demonstrated similar phenotypes, in the present study, we performed inhalation exposure to better model human-like pharmacokinetics. E-Cig aerosol exposure (inhalation) in ad lib mice delivers plasma nicotine levels relevant for E-Cig users in humans [35,36]. Given the increasing prevalence of E-Cig uses and obesity among youth and young adults, our two-hit model is a relevant experimental context that closely represents human behaviors. The age of the mice was also chosen to be comparable to young adults who use E-Cig [4].

The current findings demonstrate the adverse cellular and molecular effects of E-Cig (containing 2.4% nicotine) exposure on the skeletal muscle, especially in the presence of HFD (a two-hit model). In HFD-fed mice, E-Cig 2.4% produced stress and metabolic changes in soleus: (1) impaired lipid handling evidenced by reduced levels of p-AMPK and p-ATGL; (2) increased oxidative stress evidenced by increased levels of HO-1, reduced levels of SOD1/2, and increased levels of p-p38MAPK; (3) reduced autophagy flux, evidenced by reduced levels of LC3-II and LC3-II/LC3-I ratio and p62 accumulation; and (4) damage in mitochondrial structure by TEM. The absence of significant effects on the levels of phospho-AMPK and triglyceride accumulation suggests that the two-hit model (E-Cig and HFD) appears essential, consistent with our earlier findings, where the combination of HFD and nicotine injections induced similar adverse effects in the liver [37], heart [38], and gastrocnemius muscle, whereas nicotine injections on NCD had no effects [26].

The metabolic consequences of nicotine include increased plasma-free fatty acids (FFAs) [7,26,39], as reported by our laboratory. The apparent interaction between E-Cig and HFD is the increase in plasma-FFAs [8,39]. Pharmacologic suppression of lipolysis with acipimox mitigated the skeletal muscle injuries of nicotine-injected mice [26], supporting an FFA-mediated mechanism [26]. How does HFD exacerbate the E-Cig 2.4% effects? Our data suggest that the HFD stress amplifies E-Cig-induced injury, consistent with a two-hit model. Prior and current findings suggest that nicotine-driven lipolysis (probably from adipose tissue) elevates FFAs, which overload skeletal muscle lipid handling, contributing to the metabolic and structural changes that we observed. In contrast, E-Cig + NCD showed no significant changes in key endpoints, underscoring that diet-induced obesity is required for the full pathological phenotype.

Mechanistically, AMPK plays an essential role in maintaining skeletal muscle metabolism [27] by regulating diverse processes such as mitochondrial integrity or autophagy activity [27]. We observed a significant reduction in phosphorylated AMPK and its downstream target ATGL, a crucial lipolytic enzyme [29], in the skeletal muscle of mice exposed to E-Cig 2.4% and HFD, leading to triglyceride accumulation in the HFD groups. Our findings are consistent with the findings in ATGL^-/-^ mice, which have altered lipid metabolism, including intracellular lipid accumulation, reduced lipolysis, and fatty acid oxidation [40], and worse exercise performance [40], as well as with the findings in mice with E-Cig exposure [23,24,25].

On the other hand, oxidative stress is a common feature of nicotine when delivered parenterally or via E-Cig 2.4% exposure in different tissues, including the skeletal muscle, as we reported previously [7,39]. Our findings provide insight into the cellular mechanism of E-Cig 2.4% exposure, including increased oxidative stress, evidenced by increased levels of the cytoprotective enzyme, HO-1 [30], and reduced levels of the antioxidant enzymes, mitochondrial SOD2 and the cytoplasmic SOD1, to neutralize the oxidative stress [31]. Oxidative stress activates stress-related pathways, including the phosphorylation of p38 MAPK [41,42], which is involved in pathological processes such as the stimulation of protein degradation [41,42] and fibrosis of the skeletal muscle [43].

Reduction in the LC3-II/LC3-I ratio and accumulation of p62 levels suggest that E-Cig (2.4% nicotine) cause a decrease in the degradation through autophagy [33], a homeostatic cellular mechanism that is highly controlled for the maintenance of healthy cells. In the skeletal muscle, both increased and decreased autophagy are harmful [44]. When autophagy is reduced, cells fail to efficiently degrade and recycle damaged proteins and organelles, leading to their accumulation and increased oxidative stress, thereby compromising cellular homeostasis [44]. Although our results suggest decreased autophagy, further studies are necessary, such as flux assays using bafilomycin [33].

The critical result of the E-Cig and HFD effects is the mitochondrial structural damage as (1) the reduced AMPK activity reduces mitochondrial biogenesis [45]; (2) increased levels of oxidative stress [46] and p-p38MAPK [47] promote mitochondrial dysfunction; and (3) the reduced levels of autophagy result in the accumulation of damaged mitochondria [46]. Since the primary source of energy in the skeletal muscle is the mitochondria, which promotes lipid oxidation, the damage that we observed could have relevant consequences for E-Cig users.

Little is known about the effects of E-Cigs on skeletal muscle function, as they are a relatively new product. Preliminary data on humans showed that after bike exercise, E-Cig users demonstrated reduced endurance capacities and earlier onset of fatigue during performance testing and higher levels of lactate compared to non-smokers [23], suggesting a possible reduction in skeletal muscle function. In the murine model, Chen and colleagues showed that female mice exposed for 14 days to E-Cigs undergo a reduction in physical capacities for swimming and grip strength [25]. Nogueria and colleagues also reported mice with a reduction in exercise performance on the treadmill after 16 weeks (4 months vs. our 3 months plus HFD) of E-Cig exposure [24]. Our E-Cig exposure system differs from previous reports that used refillable tank devices and NCD alone. Specifically, our E-Cig model provides a robust analysis of the chemistry in important conditions, such as nicotine chemistry and pH [48]. By using cartridge-based Blu PLUS^TM^ [24], we ensured dose consistency and achieved human-like nicotine pharmacokinetics (plasma levels of nicotine 23.4 ± 3.3 ng/mL; cotinine 254.1 ± 42.1 ng/mL concentration) [35,36], thereby improving translational relevance.

Our experiments were performed in the soleus muscle, which is an oxidative muscle with a high content of mitochondria composed of fiber type I (oxidative) and IIa (oxidative–glycolytic) fibers [49]. Oxidative fibers support superior endurance performance, whereas a shift toward glycolytic (type IIb) fibers diminishes exercise capacity [49]. High mitochondrial damage in the E-Cig 2.4% nicotine group impaired capacities [24,25]. However, the effects of E-Cigs on the proportion of muscle fiber types and exercise endurance are still not clear.

We also noted the changes in response to E-Cig 0% nicotine, including reduced phosphorylation of ATGL, reduced levels of LC3-II, increased levels of HO-1, and decreased levels of SOD2. We speculate that some detrimental consequences may arise from the compounds, such as acetaldehyde, acrolein, formaldehyde, and other aldehydes, produced from propylene glycol and glycerol during vaping [50,51] and/or their decomposition chemicals during the heating of the E-Cig, other than nicotine [52]. Moreover, components such as acrolein have been shown to directly target the skeletal muscle [53]. All these chemicals are known toxic molecules and likely add to the negative effects of nicotine.

In this study, we used male mice since studies suggest that males initiate and purchase more E-Cigs than females [54]. Sex-specific effects of E-Cig have been previously reported [54], including greater asthma susceptibility, higher cardiopulmonary immune responses in males, and higher risks for strokes in females [54]. Thus, we plan to include female mice in future studies. Although Blu PLUS^TM^ E-Cig was a popular brand at the start of our study, it did not receive FDA approval and is no longer commercially available [55]. Thus, in our future studies, we plan to use currently available FDA-approved E-Cigs, such as Juul, which has higher nicotine levels [56].

Finally, the adverse consequences of E-Cigs and HFD on the skeletal muscle have important implications for the long-term health of E-Cig users, and this is a public health concern as muscle weakness and atrophy (sarcopenia) are associated with the development of chronic disease [57] and mortality [21]. The skeletal muscle is fundamental for the prevention of metabolic chronic diseases; the metabolic function of the skeletal muscle needs to be carefully studied. Our research opens up new concerns about the adverse effects of the E-Cig, providing a new opportunity for reducing the perceived appeal of E-Cig use. Furthermore, the findings could be used for educating and formulating public policy to prevent/reduce the use of the E-Cig.

## 4. Materials and Methods

### 4.1. Mice

Animal handling and experimentation were performed in accordance with the recommendation of the current National Institutes of Health guidelines and approved by the Charles R. Drew University and Lundquist Institute Animal Care and Use Committees (IACUCs). This study was reported following the ARRIVE 2.0 Essential 10 guidelines (http://arriveguidelines.org, accessed on 18 November 2025) [58]. Adult (12-week-old) male C57BL/6J mice (22–24 g), purchased from Taconic Farms (Germantown, NY, USA), were housed (4–5 per cage) in a standard animal facility under controlled temperature (22 °C) and photoperiod (12 h light and 12 h dark cycle) with food and water ad libitum. Animals were housed in ventilated polysulfone cages with standard bedding. Male mice were used to maintain consistency with our previous studies for comparison. Sex-relevant effects of E-Cigs and the effects in female mice are discussed in the Section 3.

Mice were fed an NCD (18% of calories from fat consisting of 18.1% of protein, 45% of carbohydrate, and 6.2% of fat; 7013; Teklad Diet, Madison, WI, USA) or HFD (60% of calories from fat consisting of 26.2% of protein, 26.3% of carbohydrate, and 34.9% of fat; D12079B; Research Diets, New Brunswick, NJ, USA). Mice on both diets were exposed to control (saline aerosol, *n* = 5), E-Cig 0% (Blu PLUS^TM^ E-Cig with 0% nicotine classic tobacco flavor, *n* = 9), or E-Cig 2.4% (Blu PLUS^TM^ E-Cig with 2.4% nicotine classic tobacco flavor, *n* = 10). The E-Cig 0% nicotine group was included to isolate the effects of nicotine from other vapor components. While non-nicotine constituents such as propylene glycol, glycerol, and flavoring aldehydes are known to induce oxidative stress [52], this group allows for a comparison to be made between nicotine-containing and nicotine-free exposure within the same delivery system. The number of animals per group was based on the availability of chambers for simultaneous and safe exposure of animals under uniform aerosol conditions and is consistent with similar studies in the field. We adhered to the principle of reduction (3Rs) while ensuring biological and analytical relevancy to detect meaningful differences in the primary endpoints of interest.

Mice were exposed 12 h/day for 12 weeks using our well-established chronic intermittent exposure protocol with E-Cig aerosol generation and rodent exposure system, as described previously [35]. Briefly, the software and hardware controlled the timing of E-Cig exposure. Puff duration was set at 4 s. Six puffs per vaping episode with an inter-puff interval of 26 sec and one vaping episode every 30 min were set. Mice were exposed to intermittent E-Cig aerosol (24 vaping episodes) for 12 h (“on”) per day. During the 12 h “off” period, mice were returned to their home cages without any aerosol exposure. This protocol, after 12 h of E-Cig exposition, produces nicotine pharmacokinetics similar to those of human heavy vapers [35]. After a staggered 12 h of exposure, the final exposure ended at 08:00, 09:00, 10:00, and 11:00 a.m. Within 1 h of the last E-Cig exposure under anesthesia, the mice were sacrificed with 5% isoflurane/CO_2_. The soleus, tibialis anterior, and gastrocnemius muscles were carefully dissected from each mouse, quickly snap-frozen in liquid nitrogen, and stored at −80 °C. The soleus muscle was used for analysis (Supplementary Figure S1).

### 4.2. Western Blot Analyses

For Western analysis, the tissues were lysed and homogenized in Tris-EDTA buffer (50 mM Tris, 10 mM EDTA, pH 8,3) containing a cocktail of protease and phosphatase inhibitors (Thermo Scientific, Hampton, NH, USA), as previously described [59]. The proteins (60–100 μg) were separated on 10–12% SDS-PAGE in TRIS-glycine-SDS buffer (Fisher Scientific, Hampton, NH, USA) with 100–120 V and transferred to a nitrocellulose membrane (Bio-Rad, Hercules, CA, USA) for 1 h at 300 mAmp at 4 °C in TRIS-glycine buffer (Bio-Rad, Hercules, CA, USA). The membranes were stained with Ponceau S solution (Sigma Aldrich, St. Louis, MO, USA) for 5 min at room temperature, rinsed with distilled water, and blocked in 0.1% Tween-20, TRIS buffer saline pH 7.4, and 5% non-fat milk (blocking solution) for 1 h at room temperature. The membranes were probed with the following antibodies—rabbit polyclonal p-AMPK (1:1000) (2535; Cell Signaling Technology, Beverly, MA, USA), rabbit polyclonal total AMPK (1:1000) (2532; Cell Signaling Technology), rabbit polyclonal p-ATGL (1:1000) (ab135093; Abcam, San Francisco, CA, USA), rabbit polyclonal HO-1 (1:2000) (ab13243; Abcam, USA), rabbit polyclonal LC3B (1:3000) (ab51520, Abcam, USA), mouse monoclonal phospho-p38 MAPK (p-p38) (clone D8, sc-7973; Santa Cruz Biotechnology, Santa Cruz, CA, USA), rabbit polyclonal SOD1 (1:500) (sc-11407; Santa Cruz Biotechnology, USA), rabbit polyclonal SOD2 (1:500) (sc-30080; Santa Cruz Biotechnology, USA), or rabbit polyclonal β-Actin (1:4000) (ab8227; Abcam, USA). β-Actin was used as a loading control. All antibodies were incubated overnight at 4 °C with constant shaking. The membranes were rinsed in TBS-Tween 20, incubated with anti-mouse or anti-rabbit IgG secondary antibody (Abcam, USA), and rinsed in TBS-Tween 20, and protein was visualized by chemiluminescence using ECL detection kits (Thermo Fischer Scientific, Waltham, MA, USA) and the imaging system LI-COR Odyssey^®^ XF (LI-COR, Lincoln, NE, USA). Band intensities were quantified using ImageJ 1.54g software (National Institutes of Health, Bethesda, MD, USA).

### 4.3. Skeletal Muscle Triglyceride Quantification

Frozen skeletal muscles were homogenized using NP-40 buffer with proteases and phosphatase inhibitors (Sigma Aldrich, St. Louis, MO, USA). We quantified the triglycerides using the commercially available triglyceride colorimetric assay kit (Cayman Chemical, Ann Arbor, MI, USA) in the skeletal muscle, following the manufacturer’s instructions. The samples were normalized to protein concentration in each sample. Protein quantification was performed with the BCA assay (Thermo Scientific, Hampton, NH, USA) following the manufacturer’s instructions and quantified using a plate reader, Biotek 800TS (Agilent, Santa Clara, CA, USA).

### 4.4. Transmission Electron Microscopy Analyses

Transmission electron microscopy (TEM) was performed to assess mitochondrial ultrastructure. Soleus skeletal muscles, fixed in 2.5% glutaraldehyde (Sigma Aldrich, St. Louis, MO, USA), were post-fixed in 1% osmium tetroxide, dehydrated in a graded series of ethanol, and embedded in Epon 812, as described previously [26]. Thin sections from embedded muscle tissues were cut with an LKB ultramicrotome, stained with uranyl acetate and lead citrate, and examined with a Hitachi electron microscope (Hitachi, Indianapolis, IN, USA).

### 4.5. Statistical Analyses

Statistical analyses were performed using Prism 9.1.0 software (Prism^®^, GraphPad^®^ Software, Inc., San Diego, CA, USA). Data were shown as mean ± standard error of the mean (S.E.M.). Multiple comparisons were performed with one-way ANOVA, with Holm–Sidak’s method as a post hoc test. Differences were considered significant if *p* < 0.05.

## 5. Conclusions

**Figure 5:**
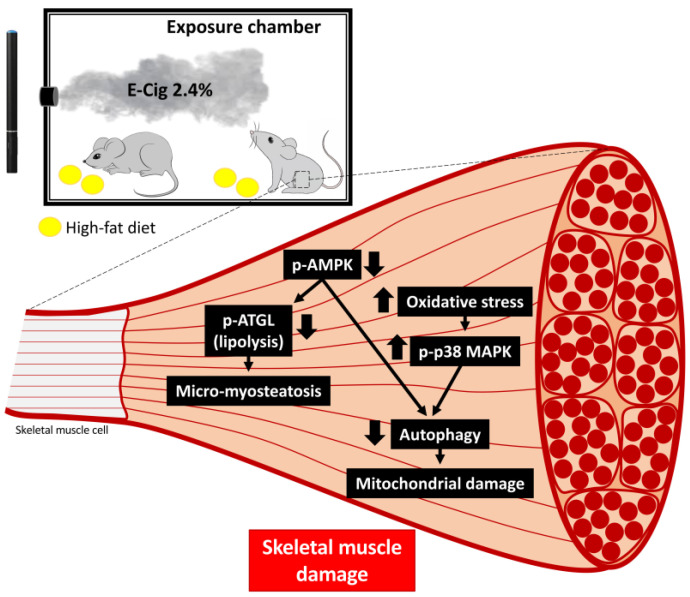
Proposed model illustrating the cellular and molecular mechanisms of E-Cig-induced skeletal muscle damage. The skeletal myocytes of mice exposed to E-Cig 2.4% show dephosphorylation of AMPK and ATGL, with increases in micro-myosteatosis. E-Cig 2.4% increases oxidative stress with activation of p-p38 MAPK. These imbalances of metabolism (AMPK) and stress (p38 MAPK) pathways lead to autophagy deregulation, resulting in accumulation of damaged mitochondria.

In summary, HFD-fed mice exposed to E-Cig 2.4% nicotine had cellular detrimental effects on the skeletal muscle, including disrupted AMPK signaling, lipid accumulation, oxidative stress and stress signaling, autophagy reduction, and mitochondrial damage (Figure 5). The adverse consequences of E-Cig and HFD on the skeletal muscle have important implications for the long-term health of E-Cig users, and this is a public health concern as muscle weakness and atrophy (sarcopenia) are associated with the development of chronic disease [57] and mortality [21]. These findings also highlight that diet–vaping interaction may increase risks for muscle dysfunction in metabolically vulnerable populations, adolescents, and young adults, providing a great tool for educating and formulating public policy for the prevention of and/or reduction in E-Cig use.

## Data Availability

The original contributions presented in this study are included in the article/Supplementary Materials. Further inquiries can be directed to the corresponding author.

## Supplementary Materials

The following supporting information can be downloaded at: https://www.mdpi.com/article/10.3390/ijms262311491/s1.

## Abbreviations

AMPK, AMP-activated protein kinase; ATGL, adipose triglyceride lipase; E-Cig, electronic cigarette; HFD, high-fat diet; HO-1, heme oxygenase-1; IMCL, intramyocellular lipid; ip, intraperitoneally; LC3-I/II, microtubule-associated protein 1 light chain 3 (I = cytosolic; II = lipidated/autophagosome-associated); MAPK, mitogen-activated protein kinase; NCD, normal chow diet; p-, phosphorylated; p62/SQSTM1, sequestosome-1; SOD1/2, superoxide dismutase 1/2; TEM, transmission electron microscopy.
