# Supplementary material for: Electronic Cigarette Exposure Induces Adverse Cellular Alterations in Skeletal Muscle in Male Mice Subjected to a High-Fat Diet
Source: Int J Mol Sci. 2025 Nov 27;26(23):11491. doi: 10.3390/ijms262311491 (PMC12691771; doi:10.3390/ijms262311491)
Supplement: Supplementary file 1 [file ijms-26-11491-s001.zip › ijms-3886931-supplementary.pdf]

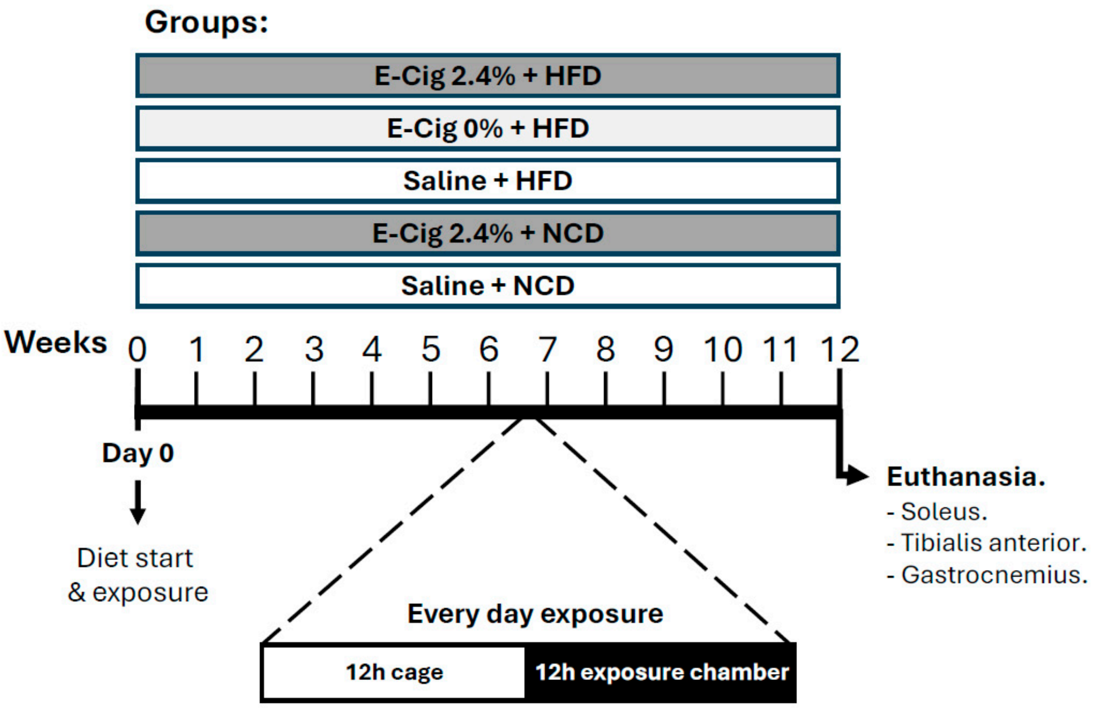

Supplementary Figure S1. Schematic diagram of experimental design. Male C57BL/6J mice were exposed to an E-Cig chamber (exposure chamber) with saline solution, E-Cig 0% nicotine, or E-Cig 2.4%, and fed with NCD or HFD for 12 weeks. Aerosol exposure and diets began and ended at the same time. Every day, exposure was 12 hours in the exposure chamber and 12 hours in their cages (dashed-line insert). At the end, we collected the soleus, tibialis anterior, and gastrocnemius.

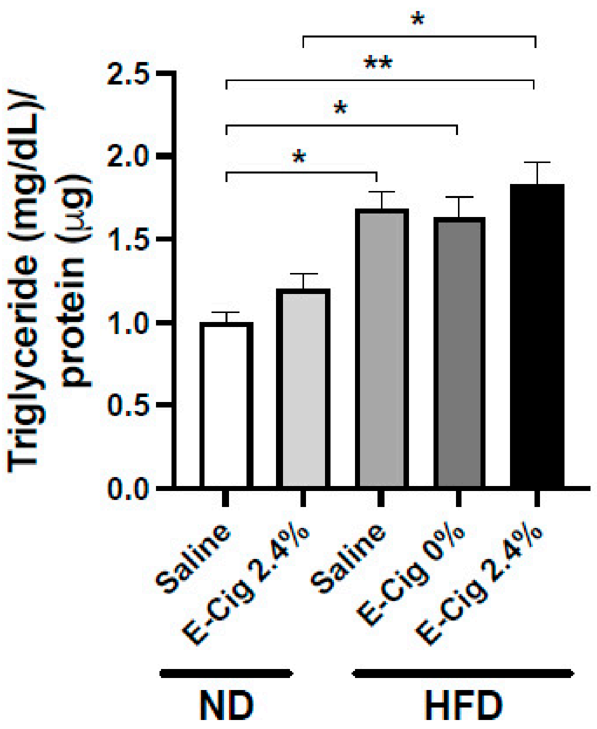

Supplementary Figure S2. Quantification of skeletal muscle triglyceride levels. Triglycerides were quantified as explained in the method section. Values are shown as mean ± S.E.M. (fold-change relative to the Saline + NCD group). **p* < 0.05, ** *p* < 0.01.
